# Obituary

**Published:** 1868-12-15

**Authors:** 


### OBITUARY.

DIED, in this place, on Tuesday, November 24th, after a brief illness of congestion of the stomach and bowels, Henry W. Davis, M.D., in his 42nd year.

Dr. Davis began the study of medicine in Carlisle, Ind., with his uncle, Dr. Helms, and concluded while attending Medical College, in Baltimore, as private student of Prof. Samuel Chew, M.D., and graduated at the same College, in the spring of 1852. In 1854 he located in this place, and began the practice of his profession (associated with Dr. John Tenbrook), which he pursued, with great satisfaction and credit to himself and his patrons, until April, 1861.

On the breaking out of the Rebellion he entered the service as a private, in Company E, 12th Illinois Infantry Volunteers. Shortly after reaching Springfield he went before the State Board of Medical Examiners, and was made a surgeon of volunteers, and immediately after became a member of the Board, which position he held until, by his request, he was made Surgeon of the 18th Regiment Illinois Infantry Volunteers, with which he served until made Assistant Surgeon U. S. Volunteers, and assigned as Inspector of the 16th Army Corps.

In 1865 he was promoted to Surgeon U. S. Volunteers, and assigned to duty as Medical Director, Department West Kentucky, with headquarters at Paducah, where he continued until the close of the war.

Since Dr. Davis became a citizen of this State, he has been notably identified with medical organizations of county, district, and State, and his name is recorded in each as the author of valuable contributions to their medical archives.

From the effects of a dissection wound, received while on duty at Little Rock, Ark., he was disabled from engaging in the general practice of medicine, but he gave his attention to surgery, in which he excelled, and to which he was specially devoted.

Few men enjoyed more of the confidence and admiration of his associates than Dr. Davis, and none died more lamented for his professional, literary, and social accomplishments.

PARIS, ILL., 1868.

